# Fitting multilevel models in complex survey data with design weights: Recommendations

**DOI:** 10.1186/1471-2288-9-49

**Published:** 2009-07-14

**Authors:** Adam C Carle

**Affiliations:** 1 Department of Psychology, University of North Florida, 1 UNF Drive, Jacksonville, FL, 32224, USA

## Abstract

**Background:**

Multilevel models (MLM) offer complex survey data analysts a unique approach to understanding individual and contextual determinants of public health. However, little summarized guidance exists with regard to fitting MLM in complex survey data with design weights. Simulation work suggests that analysts should scale design weights using two methods and fit the MLM using unweighted and scaled-weighted data. This article examines the performance of scaled-weighted and unweighted analyses across a variety of MLM and software programs.

**Methods:**

Using data from the 2005–2006 National Survey of Children with Special Health Care Needs (NS-CSHCN: *n *= 40,723) that collected data from children clustered within states, I examine the performance of scaling methods across outcome type (categorical vs. continuous), model type (level-1, level-2, or combined), and software (Mplus, MLwiN, and GLLAMM).

**Results:**

Scaled weighted estimates and standard errors differed slightly from unweighted analyses, agreeing more with each other than with unweighted analyses. However, observed differences were minimal and did not lead to different inferential conclusions. Likewise, results demonstrated minimal differences across software programs, increasing confidence in results and inferential conclusions independent of software choice.

**Conclusion:**

If including design weights in MLM, analysts should scale the weights and use software that properly includes the scaled weights in the estimation.

## Background

### Introduction

Multilevel models (MLM) offer analysts of large scale, complex survey data a relatively new approach to understanding individual and contextual influences on public health. Complex sampling designs organize populations into clusters (e.g., states or counties) and then collect data *within *the clusters. For example, a survey may first identify clusters (e.g., all counties within an area), sample the clusters (i.e., select some but not all of the counties), and then select units within the clusters (e.g., people within a county). These sampling plans result in non-independent data. People within the clusters tend to be more similar to each other than they are to people in other clusters. This can result in biased standard errors and parameters when analyzed using analytical techniques that do not take the clustered nature of the data into account. This, in turn, can lead to increased Type I errors, causing analysts to incorrectly reject null hypotheses. [1-10] Analysts have traditionally used techniques that treat the clustered nature of complex survey data as a nuisance by adjusting the standard errors for the sampling design. This method delivers correct standard errors and properly accounts for non-independence. However, it fails to allow analysts to examine the amount of between-cluster variance unaccounted for by predictors included in the model. [11-14] As public health research becomes increasingly interested in contextual influences on health (e.g., state or neighborhood level influences on health), analysts need to adopt methods that allow investigations of variance within and between clusters. [14,15]

MLM offer a unique solution to this problem. They take into account the clustered nature of the data *and *they allow analysts to investigate sources of variations within and across clusters. Thus, they allow analysts to describe which variables predict individual differences, they allow analysts to describe which variables predict cluster level differences (e.g., state level differences), and they allow analysts to explore variation across and within clusters. Moreover, because MLM explicitly model the clustered nature of the data, MLM can correctly estimate standard errors and lead to more accurate inferential decisions. [16,17] Traditional programs for analyzing complex survey data (e.g., SUDAAN)[18] use program commands to correct the standard errors for the sampling design, but treat the sampling design as a nuisance. In MLM, one expresses the sampling design as part of the equations in the model (Appendix C presents a series of MLM equations), rather than expressing the design outside the model. [17] In this way, analysts can investigate variance within and across clusters.

However, despite the unique contribution MLM can make to understanding public health, [11] they have not been widely adopted by analysts using complex survey data. Limited adoption occurs for several reasons. First, complex survey designs often involve unequal selection probabilities of clusters and/or people within clusters. The surveys include design (sampling) weights to account for unequal selection probabilities. MLM analyses that incorporate sampling weights use a pseudomaximum likelihood estimation approach. [19] Because the level-1 and level-2 weights appear in separate places within the pseudomaximum likelihood estimator function, it is not sufficient to know the product of the level-1 and level-2 weights. [20] Thus, one must take special care to include design weights. Yet, until recently, few guidelines existed for incorporating design weights into multilevel models. Second, software programs have only begun to correctly include design weights in MLM estimation. Third, little work with empirical (as opposed to simulated) data has compared and contrasted different methods for handling design weights. And, fourth, few explorations have compared the performance of the methods across the major software programs for MLM that allow incorporation of design weights.

In this paper, I take a non-mathematical approach and seek to address these issues. First, I briefly summarize the results of simulation studies and suggest a current best practice recommendation with regard to handling design weights in MLM. Second, I compare and contrast the results of different methods for incorporating sampling weights across a series of MLM using continuous and categorical outcomes and level-1 (individual) and level-2 (cluster) predictors in empirical data across three of the main MLM software programs: Mplus,[21] MLwiN,[22] and GLLAMM. [23] Third, in Appendix A, I provide example weight-scaling code so that readers can replicate and extend these findings in their own data. Finally, I conclude with some comments on the strengths and weaknesses of each software program and summarize some of the remaining methodological issues.

### Incorporating Design Weights in Multilevel Models

#### Summary of Simulation Work

Complex sampling designs regularly incorporate unequal selection probabilities. Failing to account for this aspect of the design in the standard MLM can lead to biased parameter estimates. [10,20] Thus, while the standard MLM can properly estimate parameters and standard errors in clustered data that resulted from equal probability sampling,[16,17] the standard MLM may lead to biased estimates when employed in samples that include unequal probability of selection. To rectify this problem, analysts have recommended incorporating design weights in the likelihood function. [1-10,20,24] The design weights account for unequal selection probabilities. As numerous authors have discussed, though, when estimating MLMs, to properly include design weights in the likelihood function requires scaling the weights. One cannot simply use the "raw" weights. [1-10,20,24] To address this, numerous scaling methods have been proposed[1-3,9,10] and analysts have undertaken simulation work to examine the behavior of the scaling methods in simulated data in attempt to identify a scaling method that provides the least biased estimates in most situations[1-3,10,20] (In an effort to focus on analytical application, I do not review the mathematics associated with this work. For further discussion on the mathematical derivations associated with this work, readers should consult Asparouhov [1-3] or Rabe-Hesketh and Skrondal[10]).

Some consistent themes result from this work. First, simulations indicate that most scaling methods consistently provide better estimates than using *unweighted *analyses. [1-3,10] Second, two scaling methods have emerged that appear to provide the least biased estimates in general. [1] Method A,[1] scales the weights so that the new weights sum to the cluster sample size. Method B,[1] scales the weights so that the new weights sum to the effective cluster size. These methods have received various labels in the literature. For consistency, I use Asparouhov's [1] labels. Third, no gold standard scaling method has emerged. This transpires because the simulation studies all show that that various features of the design *and *data can affect a scaling method's adequacy. [1-3,10,20] For example, as cluster sizes increase, the estimates generally become less biased. [1,9,10] This suggests that with sufficiently sized clusters, an analyst may worry less about scaling the weights. Yet, because the number of clusters, the size of the clusters, the type of outcome (categorical vs. continuous), the size of the correlation between the outcome and design weight, and the design weights' informativeness can all independently and jointly affect a scaling method's results, a priori scaling method decisions can be difficult. [1]

Fourth, the simulations point to a need for *some type *of scaling if using weights, especially with small cluster sizes. If one cannot scale the weights and include them properly in the estimation, analyzing the data *without *weights provides the next best option. Including the weights but failing to scale them (i.e., including them as "raw" weights results in biased parameters and standard errors, especially with small cluster sizes. [10] Fifth, as these researchers note,[10] few publicly available data include weights for each level of analysis. Rather, publicly available data usually include a single overall level-1 weighting variable that incorporates level-2 design issues. This confounding of level-1 (individual) and level-2 (cluster) design issues in a single weight can result in biased estimates. [10] Thus, along with choosing the appropriate scaling method, one must also decide whether to use the "level -1" weights to estimate higher level weights or whether to leave the higher levels unweighted. However, the choice of the scaling of the level-2 weights will not influence parameter estimates or the standard errors associated with these estimates if level 2 corresponds to the highest level of the model and the same scale factor applies to all units. [3,10] Finally, different estimation procedures and convergence criteria may lead to dissimilar results even when using identical scaling methods. [1-3]

#### Recommendations

Given that no study will include all possible manifestations of complex survey designs and relations among the data, it is impossible to disentangle these issues and arrive at a single gold standard. [1-3] Thus, based on the results of the simulation work,[1,10] one should *not *use a *single *scaling method. Rather, analysts should fit the MLM using both scaling methods (A and B) and unweighted data. Then, one should compare the results across methods. To the extent that the inferential decisions converge, analysts gain confidence in the results. When the inferential decisions diverge, analysts should conduct a detailed analysis that includes Monte Carlo simulations to determine which method provides the least biased estimates. [1,10] Additionally, the simulation work suggests that, for point estimates (e.g., intercepts, odds ratios, etc.), method A will often provide the least biased estimates. [1] Thus, analysts who wish to discuss point estimates should report results based on weighting method A. For analysts more interested in residual between-cluster variance, method B may generally provide the least biased estimates. [1] For variance-covariance discussions, then, analysts should report results based on method B. However, as cluster sizes increase (*n *> 20), method A appears to increase its advantage,[1] though bias decreases substantially for all methods as cluster sizes become sufficiently large. [1,9,10] Thus, when working with cluster sizes larger than *n *= 20 and a concern that insufficient cluster size may lead to biased estimates, analysts may wish to report method A's results.

This suggestion, using multiple scaling techniques, points to an important issue. To properly conduct MLM with complex survey data and design weights, analysts need software that can include weights scaled outside of the program and include the "new" scaled weights without automatic program modification. Currently, three of the major MLM software programs allow this: Mplus (5.2)[21], MLwiN (2.02),[22] and GLLAMM. [23] Unfortunately, neither HLM[25] nor SAS[26] can do this. One cannot include pre-scaled weights in HLM analyses. Likewise, SAS MLM procedures treat the weights as frequency weights rather than sampling weights. Thus, they do not properly include the weights in the likelihood estimation (though Grilli and Pratesi[20] developed a relatively complicated method to "trick" SAS NLMIXED into properly handling weights under some conditions (e.g., models with no more than two levels)). Additionally, one cannot fit MLM using SAS Survey procedures. Thus, one should not generally use either of these programs to fit MLM in complex survey data with design weights.

In sum, simulation work suggests that analysts should fit complex survey data with design weights using a variety of scaling methods (including unweighted) and compare the results of these methods. However, little work provides a comparison of the different scaling methods in real data across a variety of MLM (e.g., continuous vs. categorical outcomes, level-1 predictor models, level-2 predictor models, and models including level-1 and -2 predictors simultaneously) and software programs. Thus, it remains unclear whether real data will reflect simulation work. In the next section, I address this issue. I use data from the 2005–2006 National Survey of Children with Special Health Care Needs[27] and fit a series of MLMs using Mplus, MLwiN, and GLLAMM and compare and contrast the estimates and their standard errors across the programs and scaling methods.

## Methods

### Comparing Scaling Methods and Software in Real Data

To examine the performance of the various scaling methods, I fit two series of MLM. I chose these models because they represent the basic models presented by major texts on MLM (e.g., Raudenbush and Bryk[16]), the models form the building blocks for more complicated models, and because the models in each series represent typical types of models analysts would explore in MLM. [16,28] I used publicly available data from the 2005–2006 National Survey of Children with Special Health Care Needs (NS-CSHCN: downloadable at ftp://ftp.cdc.gov/pub/Health_Statistics/NCHS/slaits_cshcn_survey/2005_2006/Datasets/), sponsored by the Maternal and Child Health Bureau (MCHB) and conducted by the National Center for Health Statistics (NCHS). Within each state and Washington DC (hereafter state includes Washington DC), this survey used random digit dialing and collected data on approximately 750 children with special health care needs (CSHCN). It represents a "classic" two level design. CSHCN (level-1) nested within states (level-2). Given that the survey design specified approximately equal sample sizes for each state (*n *≅ 750 for each state), children in smaller states had a greater probability of selection. Likewise, in households with multiple children, one randomly selected CSHCN served as the subject. Thus, CSHCN in smaller families had a greater probability of selection. Level-1 design weights account for these unequal selection probabilities, adjusted for other design issues (e.g., nonresponse), and weight the data to make it representative of the CSHCN in the US. The NS-CSHCN sampled each state with certainty. Thus, states were not selected with unequal probability and do not need weights. As described, the level-1 weights account for unequal probability of selection given different population sizes within states. Thus, I left level-2 unweighted. See Blumberg et al. [27] for complete details.

The first series of MLM I estimated examines a continuous outcome (the number of months CSHCN go without insurance) as a function of a level-1 predictor (family income relative to poverty level, hereafter labeled simply "family income") and a level-2 predictor (the proportion of families in the state with an income no greater than twice the US federal poverty level (i.e., 200% poverty level), here after labeled simply "proportion of families in poverty"). The second series of MLM examines a categorical outcome (whether a CSHCN went uninsured at any time in the previous 12 months) as a function of a level-1 predictor (family income) and a level-2 predictor (proportion of families in poverty). For both series, I fit six models: 1) an unconditional model, 2) a level-1 predictor only model specifying the level-1 slope as fixed, 3) a level-1 predictor only model that allowed the level-1 slope to vary across the states (level-2), 4) a level-2 only predictor model, 5) a model including level-1 and -2 predictors but no cross-level interaction, and, 6) a model including level-1 and -2 predictors and a cross-level interaction. For each series of analyses (continuous and categorical), the unconditional (empty) model examines whether the outcome (average number of months uninsured or odds of going without insurance) varies across states. The level-1 only predictor model asks whether family income predicts the outcome, while the level-2 predictor only model investigates whether the proportion of families in poverty in a state affects the outcome. The model including level-1 and level-2 predictors investigates the contributions of level-1 and level-2 predictors simultaneously, but does not include a cross-level interaction. Among other questions, it asks whether a relationship between family income and the outcome exists, controlling for the effects of the proportion of families in poverty in the state. The final model investigates the level-1 and level-2 predictors simultaneously and includes a cross-level interaction. This model asks several questions as well, including whether the relationship between family income and months without insurance differs according to the proportion of families in poverty in a state. For each series, all models allowed the intercept to vary across the states. Appendix C presents traditional MLM equations for each model I estimate.

For each series I fit the models in Mplus, MLwiN, and GLLAMM using unweighted data, scaling method A and scaling method B. For Mplus, I used MLR for both the continuous and categorical analyses. MLR delivers maximum likelihood parameter estimates with robust standard errors computed using a sandwich estimator. For categorical outcomes, MLR uses numerical integration and adaptive quadrature using 15 integration points per dimension. [21] For MLwiN, I used iterative generalized least squares (IGLS) estimation for the continuous outcome. With categorical outcomes, MLwiN utilizes a quasi-likelihood procedure that uses a Taylor series-based linearization to transform discrete responses into a continuous model that is then estimated using IGLS or reweighted IGLS (RIGLS). MLwiN uses either marginal quasi-likelihood (MQL) or predictive (penalized) quasi-likelihood (PQL) to approximate the linear transformation. [22] Rasbash et al. [22] suggest adopting a two step process employing MQL to generate starting values and PQL to arrive at the final estimates. I followed this procedure. [22] I first estimated each categorical model with 1^st ^order marginal quasi-likelihood (MQL) estimation and IGLS to obtain starting values. I then used the 1^st ^order MQL estimates as starting values for 2^nd ^order predictive (penalized) quasi-likelihood (PQL) estimation and IGLS to obtain final values. For both continuous and categorical outcomes in MLwiN, I requested robust standard errors. For all GLLAMM models, I initially used adaptive quadrature with 8 quadrature points. Consistent with Rabe-Hesketh et al.'s recommendation,[23] I subsequently refit the models using 16 quadrature points to see if I found consistent estimates. In almost all cases, the results were nearly identical. In the two instances where I obtained discrepant values, I continued increasing the quadrature points until the estimates stabilized. For all models, I requested robust standard errors, which GLLAMM computes using a sandwich estimator. [23,29] Finally, Appendix A presents the details to create these datasets and it gives code in SAS and Stata to create scaled weights using both methods and Appendix B gives the equations to scale the weights. Appendix D provides a brief description of the original weights. For complete details about the weights, readers should review Blumberg et al. [27]

## Results

First, consider the continuous results presented in Additional File 1. Across all six models, each software program converged on nearly identical results. With few exceptions, the unweighted parameter estimates and their standard errors were nearly identical across programs. The fact that the estimates did not converge perfectly across the programs may have occurred because of the relatively large cluster sizes in these data. Large cluster sizes may limit the performance of quadrature estimation, and MQL methods may work better. [23] As Rabe- Hesketh, et al. suggest,[23] analysts should check the adequacy of the quadrature points in any given situation by estimating models with increasing numbers of quadrature points. In these analyses, two models required increasing the quadrature points from 8 to 16 to achieve estimates in line with the other programs. But, again, overall, the results achieved marked similarity across programs.

With regard to the weighted analyses, across the fixed and random effects, the programs achieved nearly identical weighted results, with two exceptions. MLwiN estimated a smaller residual variance and residual variance standard error using weight method B than either Mplus or GLLAMM. Likewise, MLwiN's estimate of the slope for state poverty and its standard error diverged slightly (but consistently) from Mplus and GLLAMM at the second decimal point under all scaled weighting analyses. To investigate the source of these differences, I reran these analyses with increasingly stringent convergence criteria. In all cases, MLwiN arrived at the same estimate of the residual variance. This suggests that the discrepancy does not result from convergence issues, but results from estimation differences. In this case, the small difference led to *no *inferential differences across the software packages or weighting methods. For example, consider the final model. Across all weighting methods and software programs, one would conclude that, while variance does exist across states in the relationship between family income and months uninsured, the proportion of families in poverty in a state does not appear to affect this relationship.

For the categorical outcome presented in Additional File 2, a similar pattern resulted. Across all six models, each software program converged on similar results. Without exception, the unweighted parameters and their standard errors were similar across programs. With regard to the weighted analyses, a similar pattern resulted. Across the fixed and random effects, the programs achieved nearly identical weighted results, though MLwiN consistently estimated a marginally larger variance in the intercepts across states. Again, observed differences led to no differences in the inferential conclusions. For example, consider the final model. Regardless of weighting method or program, one would conclude that, while variance does exist across states in the relationship between family income and the likelihood that a child will go uninsured, the proportion of families in poverty in a state does not appear to affect this relationship.

Somewhat surprisingly, though the standard errors for the scaled-weighted data did range somewhat larger than unweighted analyses, the standard errors for the unweighted and scaled-weighted methods achieved remarkable consistency. This may have occurred because of the large cluster sizes in the NS-CSHCN (approximately 750 individuals in each cluster). It may also have occurred because of a relatively small intraclass correlation coefficient (a measure of the proportion of variance in the outcome attributable to clustering alone) for this outcome (e.g., 0.01 for months uninsured). It also suggests that, in these data, for these outcomes, and these predictors, the sampling weights are not particularly informative (Table 1 presents the results of single level analyses ignoring sampling design for comparison). However, this need not be the case. For situations with informative sampling weights (i.e., where the design weights correlate with the outcome), the findings could diverge greatly. [1] The weights lead to more representative population estimates, but failure to include them did not bias inferential decisions. This set of findings highlights the importance of conducting weighted and unweighted analyses. With the set, an analyst can compare differences across the approaches and evaluate the impact of different approaches on estimates and inferences. Without conducting analyses across scaling methods, it would be unclear whether the estimation process, type of outcome, or other factors biased the results. One should not simply choose a single method without exploring similarities and differences across methods.

**Table 1:** Single Level Continuous and Categorical outcome parameter and standard error estimates.

Continuous Single Level Analysis		Mplus (5.1)	Mplus (5.1)	Mplus (5.1)
Fixed Effects		Unweighted	Weight Method A	Weight Method B

*β*_0_	(Intercept for MS_UNINS)	0.453	0.429	0.433

SE		0.010	0.011	0.011

				

*β*_1_	(Slope for Family Income)	-0.088	-0.076	-0.076

SE		0.004	0.005	0.005

				

Random Effects				

	Residual Variance (Variation within States)	3.738	3.697	3.735

SE		0.096	0.110	0.111

				

Categorical Single Level Analysis		Mplus (5.1)	Mplus (5.1)	Mplus (5.1)

Fixed Effects		Unweighted	Weight Method A	Weight Method B

*β*_0_	(Intercept for MS_UNINS)	-2.513	-2.532	-2.524

SE		0.019	0.022	0.022

				

*β*_1_	(Slope for Family Income)	-0.198	-0.183	-0.183

SE		0.006	0.007	0.007

## Discussion

### Summary

In sum, the present results generally agree with simulation work. Scaled weighted findings diverged slightly from unweighted analyses, agreeing more with each other than with unweighted analyses. Also consistent with simulation work, weighted and unweighted data did not diverge greatly in general. However, while estimates and standard errors generally remained comparable, small specific changes did result. Although they did not lead to different inferential decisions in these data, they might in other data. [10] Thus, despite general comparability, analysts should conduct analyses with scaled and unweighted data as a general practice. Finally, given the relative consistency of estimates and standard errors within software programs across models the findings suggest that an analyst's software choice will depend largely on the analyst's needs relative to the program's strengths and weaknesses (summarized below) rather than on concern with regard to a given program's ability to consistenly incorporate scaled design weights. How, then, should one choose a software program?

### Software Strengths and Weaknesses

#### Strengths

Mplus has several strengths. It has tremendous flexibility and can incorporate numerous statistical models within the MLM framework well beyond "traditional" hierarchical linear and generalized linear models. One can fit factor models, latent class models, structural equation models, mixture models, latent growth curve models, and others within the MLM framework. Second, Mplus will automatically scale the weights for the user using each approach described here and Mplus allows analysts to specify weights scaled outside of Mplus. Third, Mplus offers a wide variety of estimators and link functions. Fourth, Mplus can handle both of the current recommended methods for analyzing subpopulations of complex survey data, the zero-weight approach and the multiple-group approach. [24]

MLwiN also incorporates several strengths. First, MLwiN can fit models with up to five levels, making it quite useful in multistage designs. Second, MLM has an easy point-and-click, windows-based user interface, which makes fitting MLMs easy and straightforward. Third, MLwiN incorporates several estimators. Fourth, like Mplus, MLwiN provides an automatic weight scaling feature, and, like Mplus, it allows the user to specify weights scaled outside of MLwiN. Fifth, MLwiN has numerous features available for evaluating a model's appropriateness. And, sixth, MLwiN includes several graphical features.

Finally, GLLAMM also has some distinct advantages. Like Mplus, GLLAMM offers an astounding array of models that it can fit within the MLM framework. [30] Second, GLLAMM does allow more than two cross-sectional levels. Third, GLLAMM allows the user to specify scaled weights. Finally, GLLAMM uses full pseudo-maximum-likelihood estimation for generalized linear mixed models with *any *number of levels using adaptive quadrature,[10] which may result in more appropriate standard errors, especially when working with categorical outcomes. [23]

#### Weaknesses

Despite its strengths, Mplus has some distinct disadvantages. First, it can only fit two-level cross-sectional MLM models. Although one can fit a two-level MLM and use Mplus' complex data analysis feature to properly estimate standard errors for a third level, Mplus does not allow one to investigate what predicts variation at level-3. For multistage surveys, this may be a substantial limit. Second, relative to MLwiN, Mplus offers few analytical tools for investigating model assumptions, model fit, and model diagnostics. Third, relative to MLwiN, Mplus offers few graphical tools, whether these limits outweigh its strengths will depend on the individual users needs.

MLwiN also has limits. Primarily, it cannot fit the wide variety of models that Mplus and GLLAMM can (e.g., latent class models). While MLwiN can fit some models beyond hierarchical linear and generalized linear models (e.g., multilevel confirmatory factor analyses), MLwiN does not have the full flexibility that Mplus and GLLAMM do. For users seeking to fit extremely complex models, this may be a substantial drawback. Second, MLwiN will only automatically scale the weights using method B. And third, while MLwiN does offer several estimators (e.g., iterative generalized least squares (IGLS), restricted IGLS, and Markov chain Monte Carlo (MCMC)), it does not offer as large a range of estimators as Mplus. Again, whether these weaknesses outweigh its strengths will depend primarily on the type of analysis the user expects to conduct.

Finally, GLLAMM has some noteworthy disadvantages. First, GLLAMM has well known problems with computational speed. Models that take seconds to converge in the other programs can take days (literally) to converge in GLLAMM. Aside from some minor adjustments, analysts can do little to increase GLLAMM's speed. Second, although GLLAMM has an advantage with categorical outcomes, it may be less accurate with continuous outcomes. [29,31] GLLAMM does not scale the weights for the user. Users must supply pre-scaled weights. Third, GLLAMM offers few automatic features (e.g., automatic grand or group mean centering) and diagnostic utilities. However, users familiar with STATA will find it easy to incorporate STATA commands, data manipulation, and diagnostic tools when using GLLAMM, whether these limits outweigh its benefits will depend on the user's individual needs.

#### Limitations

Although these analyses generally support the use of MLM in complex survey data with design weights, some issues remain unresolved. First, a best practice for scaling weights across multiple levels has yet to be advanced. Though Asparouhov[3] and Rabe-Hesketh and Skrondal[10] indicate that scaling level-2 weights has little practical effect, more work is needed to investigate the generality of that advice, particularly in surveys with 3 or more levels. Second, complex survey designs often employ unequal probability of selection at higher levels. For example, the National Epidemiologic Survey of Alcohol and Related Conditions[32] stratified the US into four regions. It then sampled counties within regions and people from households within counties. At both the county and household levels, unequal probability of selection occurred (e.g., some counties were more likely to be included than others). Survey organizations rarely make (or have) level-2 or beyond weights available. Some authors have suggested methods for estimating level-2 weights from level-1 weights,[17] yet more work is needed to investigate these methods' validity.

Third, MLM theoretically allow investigators to examine predictors and variance across naturally occurring clusters within complex sampling design (e.g., creating a three-level model by grouping individuals according to their county of residence using data from a two-level survey that sampled people within states). However, this flexibility may result in cross-classified data structures (e.g., hospital catchment areas overlapping states in a survey that sampled people within states). While MLM can handle cross-classified data,[16] no work has examined handling design weights in this situation.

Fourth, analysts often wish to investigate relationships within a certain subgroup. Although analysts can use interaction terms to investigate hypotheses within the specified subgroup, analysts may wish to examine a subgroup of the sample excluding other sample members entirely. For this situation, where analysts wish to investigate hypotheses among a specific subgroup only, no established guidelines exist regarding a best practice method for estimating MLM in complex survey data with design weights. When using complex surveys, one should include the entire sample in the analyses. This leaves the sample design structure whole and leads to proper estimation of variances and standard errors. However, it presents a problem when analysts would like to select a subgroup and examine a MLM for this subgroup of individuals in a sample. Analysts should *not *simply subset the data to the desired group of interest. [33,34] While some techniques have been suggested (e.g., zero-weighting[34] and multiple-group analyses[24]), the performance of these techniques in MLM with design weights needs further examination.

Finally, little work addresses esign weights. Itmissing data's remains unclear how to best handle missing data within the context of MLM, complex survey data, and design weights. Analysts might take a zero-weighting approach for missing data,[34] treating individuals with complete data as a subgroup, to address missing data. If one uses this approach, the analyst should take special care to scale the weights using the full set of weights. To evaluate the influence of missing data, analysts might conduct analyses in the full sample using selected variables for which all individuals have complete data and compare those results to identical analyses conducted on the same variable set but using the subsample of individuals with missing data on other variables of interest. Future work should explore and develop and test solutions to handle it.

While these limits highlight an array of outstanding issues that need investigation, they do not preclude analysts from employing MLM in complex survey data with design weights. Moreover, they demonstrate the need to choose a MLM program that allows flexibility with regard to design weights. Thus, as theory advances, software will not limit analyses.

#### Applied Summary Recommendations

Given the breadth of findings discussed and presented and the various strengths and weakness of each approach and software program, the reader might now wonder, "what do do in practice?" In my work, I standard approach. First, in terms of software, take the following I generally use Mplus. I do this because Mplus offers the most flexibility relative to speed. I frequently fit models that MLwiN cannot estimate (e.g., MLM multiple group structural equation models) and I rarely fit models with more than two levels (which Mplus currently cannot estimate). Analysts fitting the types of models discussed in this paper will generally find MLwiN more than meets their needs. Second, in terms of scaling the weights, I always fit the models using each scaling technique (methods A and B). I do this to examine any inferential discrepancies. If I find no inferential discrepancies, I generally report the findings from method A. I do this because I frequently work with cluster sizes larger than *n *= 20 and I am interested in both point estimates and variance-covariance discussions. If I worked with smaller cluster sizes (*n *< 20) and had an interest primarily in variance-covariance estimates, I would report the results of method B. If I had an interest primarily in point estimates, I would report the results of method A. Finally, if I encountered a model I could not estimate for some reason using scaled weighted data, I would take the following approach. I would fit a simpler model using scaled weighted and unweighted data. If I did not observe a difference in the inferential conclusions across these approaches, I would then fit the more complex model using unweighted data. However, I would include a note in my reporting of the unweighted findings highlighting that I used unweighted data and that readers should interpret the results with caution.

## Conclusion

Summarily, recent advances in statistical theory and software now allow users of complex survey data with design weights to analyze data in a MLM framework. This paper shows the utility of conducting MLM in complex survey data with design weights across a variety of scaling methods. Survey analysts should incorporate the recommendations offered in this paper and consider MLM when they seek to understand the intricate relations that may exist in complex survey data. MLM allow analysts to better understand and describe individual and contextual (cluster) level differences. This has the potential to profoundly influence public health policy. [11,14] MLM allow prevention and intervention efforts to better understand whether policies should target individual, contextual (cluster), or individual *and *contextual variables. MLM also allow investigators to better understand individual and cluster level influences on policy successes. Though some issues remain outstanding, by incorporating cutting edge techniques and software, analysts can inform efforts to understand, describe, and improve the health of a diverse world's public health.

## Competing interests

The author declares that they have no competing interests.

## Authors' contributions

Using publicly available data, I worked individually, conducted the literature searches and summaries of previous related work, undertook the statistical analyses, wrote the manuscript, conducted all revisions, and read and approved the final manuscript.

## Appendix A

### A.1 Data set creation

To recreate the data used in these analyses, one needs to merge two NCHS files and add the state-level (level-2) variable. The level-1 outcome, months without insurance, is available on the NS-CSHCN "interview" dataset as CQ905. The level-1 covariate (family income) is available on the data file NCHS entitles "multiple imputation". NCHS labels the family income variable POVLEVEL_I. For these analyses, I used a single imputation, imputation 1, as suggested by Pedlow, et al. [35]

NCHS makes both datasets available at ftp://ftp.cdc.gov/pub/Health_Statistics/NCHS/slaits_cshcn_survey/2005_2006/Datasets/. The level-2 covariate (proportion of families in poverty) is available at the US Census Bureau's website http://pubdb3.census.gov/macro/032007/pov/new46_185200_07.htm as well as Table 2. For simplicity's sake, all analyses in the current paper used individuals with complete data.

**Table 2:** Proportion of families in each state falling at or below the 200% poverty line.

Alabama	0.309	Montana	0.277
Alaska	0.226	North Carolina	0.31

Arkansas	0.362	North Dakota	0.217

Arizona	0.304	Nebraska	0.209

California	0.27	New Hampshire	0.152

Colorado	0.21	New Jersey	0.167

Connecticut	0.18	New Mexico	0.32

District of Columbia	0.288	Nevada	0.229

Delaware	0.207	New York	0.28

Florida	0.266	Ohio	0.235

Georgia	0.264	Oklahoma	0.308

Hawaii	0.185	Oregon	0.249

Iowa	0.21	Pennsylvania	0.212

Idaho	0.278	Rhode Island	0.217

Illinois	0.228	South Carolina	0.3

Indiana	0.233	South Dakota	0.234

Kansas	0.231	Tennessee	0.288

Kentucky	0.322	Texas	0.325

Louisiana	0.336	Utah	0.25

Massachusetts	0.212	Virginia	0.197

Maryland	0.154	Vermont	0.19

Maine	0.243	Washington	0.189

Michigan	0.245	Wisconsin	0.187

Minnesota	0.18	West Virginia	0.332

Missouri	0.264	Wyoming	0.209

Mississippi	0.413		

### A.2 Scaling the Weights

After creating the dataset, one needs to scale the weights. In any analyses, here or otherwise, one should scale the weights before doing *any *other data management, as the scaled weights should be based on the *entire *sample of individuals with weights.

### SAS Code

proc sort data = mlm;

      by state;

   run;

proc summary data = mlm;

   by state;

   var weight_i;

   output out = intermediate

      uss = sumsqw

      sum = sumw

      n = nj;

   run;

data mlm;

   merge mlm intermediate;

      by state;

   aw = weight_i/(sumw/nj);

   label aw = "Method A";

   bw = weight_i/(sumsqw/sumw);

   label bw = "Method B";

   run;

data mlm; set mlm; drop _freq_ sumsqw sumw nj _type_; run;

To update this code for other datasets, 1) replace "mlm" with the name of the dataset of interest, 2) replace "weight_i" with the level-1 weight from the dataset of interest, and 3) replace "state" with the level-2 cluster variable from the dataset of interest.

### Stata Code

gen sqw = WEIGHT_I^2

egen sumsqw = sum(sqw), by(STATE)

egen sumw = sum(WEIGHT_I), by(STATE)

egen nj = count(IDNUMXR), by(STATE)

gen bw1 = WEIGHT_I*(sumw/sumsqw)

gen aw1 = WEIGHT_I*(nj/sumw)

To update the Stata code for other datasets, 1) read the dataset of interest into memory, 2) replace "weight_i" with tje level-1 weight from the dataset of interest, 3) replace "state" with the level-2 cluster variable from the dataset of interest, and, 4) replace "idnumxr" with the level-1 id variable from the dataset of interest.

## Appendix B: Equations for Scaling the Weights

### B.1 Method A

### B.2 Method B

For both,  represents the scaled weight for individual *i *in cluster *j*, *w*_*ij *_the unscaled weight for individual *i *in cluster *j*, and *n*_*j *_the number of sample units in cluster *j *.

## Appendix C: Traditional MLM Equations

### C.1 Continuous Models

#### C.1.1 Unconditional model

#### C.1.2 Level-1 Predictor Only (Fixed Effect)

#### C.1.3 Level-1 Predictor Only (Fixed and Random Effects)

#### C.1.4 Level-2 Predictor Only (Fixed Effect)

#### C.1.5 Level-1 and Level-2 Predictors, No Cross-Level Interaction

#### C.1.6 Level-1, Level-2, and Cross-Level Interaction

### C.2 Categorical Models

#### C.2.1 Unconditional model

##### C.2.2 Level-1 Predictor Only (Fixed Effect)

##### C.2.3 Level-1 Predictor Only (Fixed and Random Effects)

##### C.2.4 Level-2 Predictor Only

##### C.2.5 Level-1 and Level-2 Predictors, No Cross-Level Interaction

##### C.2.6 Level-1, Level-2, and Cross-Level Interaction

#### C.3 Variance Components

In all models,

 = The variance of *e*_*ij *_(i.e., the residual variance within states).

 = The variance of *u*_0*j *_(i.e., the variance in the intercepts between states).

 = The variance of *u*_1*j *_(i.e., the variance in slopes between states).

*σ*_01 _= COV(*u*_0*j*_, *u*_1*j*_) (i.e., the covariance between the intercepts and slopes).

## Appendix D: Original Weights

This appendix briefly summarizes the methodology used to weight the 2005–2006 NS-CSHCN. Generally, the weighting scheme for the sample involved the steps below. Here, I only describe the base weights. Readers interested in more detail should consult Blumberg et al. [27].

1. Compute base sampling weight.

2. Adjustment for nonresolution of released telephone numbers.

3. Adjustment for incomplete age-eligibility screener.

4. Adjustment for incomplete CSHSCN Screener.

5. Adjustment for multiple telephone lines.

6. Raking adjustment of household weights.

7. Raking adjustment of child screener weights.

8. Adjustment for subsampling of CSHCN.

9. Adjustment for nonresponse to the CSHCN interview.

10. Raking of adjustment of the nonresponse-adjusted CSHCN interview weights.

The base weight equals the reciprocal of the selection probability of the *k*^*th *^telephone number:

*π*_*k *_= probability of selecting the *k*^*th *^telephone number in the estimation area.

*n*_*q *_= sample size in quarter *q *in the estimation area.

*N*_*q *_= total telephone numbers on the sampling frame in quarter *q *in the estimation area.

Following computation of the base weight, several adjustments followed. Blumberg, et al.,[27] describe these adjustments in detail.

## Pre-publication history

The pre-publication history for this paper can be accessed here:

http://www.biomedcentral.com/1471-2288/9/49/prepub

## Supplementary Material

Additional file 1 **Continuous outcome parameter and standard error estimates across level-1, level-2, combined-level models, weight scaling methods, and software programs**. The data provided present the multilevel results across continuous models, weight scaling methods, and software programs. Click here for file

Additional file 2 **Categorical outcome parameter and standard error estimates across level-1, level-2, combined-level models, weight scaling methods, and software programs** (Mplus estimates a threshold rather than an intercept. These differ only in sign. [21]**For presentation, I converted the threshold to a slope)**. The data provided present the multilevel results across categorical models, weight scaling methods, and software programs. Click here for file
