# Supplementary material for: Fitting multilevel models in complex survey data with design weights: Recommendations
Source: BMC Med Res Methodol. 2009 Jul 14;9:49. doi: 10.1186/1471-2288-9-49 (PMC2717116; doi:10.1186/1471-2288-9-49)
Supplement: Additional file 1 — Continuous outcome parameter and standard error estimates across level-1, level-2, combined-level models, weight scaling methods, and software programs. The data provided present the multilevel results across continuous models, weight scaling methods, and software programs. [file 1471-2288-9-49-S1.pdf]

MLM Unconditional Model		Mplus (5.1)	MLwiN (2.02)	GLLAMM (Stata 9.2)	Mplus (5.1)	MLwiN (2.02)	GLLAMM (Stata 9.2)	Mplus (5.1)	MLwiN (2.02)	GLLAMM (Stata 9.2)
Fixed Effects		Unweighted			Weight Method A			Weight Method B		
β_0	(Intercept for MS_UNINS)	0.453	0.453	0.455	0.457	0.457	0.456	0.457	0.457	0.457
SE		0.030	0.030	0.024	0.029	0.029	0.026	0.029	0.029	0.029
Random Effects										
σ^2_ε	Residual Variance (Variation within States)	3.752	3.752	3.752	3.703	3.704	3.703	3.742	2.710	3.742
SE		0.263	0.263	0.266	0.254	0.254	0.257	0.262	0.200	0.265
σ^2_0	Variance in the Intercepts Between States	0.040	0.040	0.040	0.038	0.038	0.039	0.037	0.038	0.037
SE		0.012	0.012	0.010	0.010	0.010	0.011	0.010	0.010	0.010

MLM Level-1 Predictor Only (Fixed Effect)		Mplus (5.1)	MLwiN (2.02)	GLLAMM (Stata 9.2)	Mplus (5.1)	MLwiN (2.02)	GLLAMM (Stata 9.2)	Mplus (5.1)	MLwiN (2.02)	GLLAMM (Stata 9.2)
Fixed Effects		Unweighted			Weight Method A			Weight Method B		
β_0	(Intercept for MS_UNINS)	0.453	0.453	0.455	0.430	0.430	0.430	0.430	0.430	0.430
SE		0.030	0.030	0.024	0.028	0.028	0.028	0.028	0.028	0.028
β_1	(Slope for Family Income)	-0.088	-0.088	-0.088	-0.078	-0.078	-0.078	-0.078	-0.078	-0.078
SE		0.009	0.009	0.009	0.008	0.008	0.008	0.008	0.008	0.008
Random Effects										
σ^2_ε	Residual Variance (Variation within States)	3.698	3.698	3.698	3.657	3.658	3.696	3.696	2.676	3.696
SE		0.256	0.256	0.258	0.248	0.248	0.258	0.256	0.196	0.258
σ^2_0	Variance in the Intercepts Between States	0.041	0.041	0.040	0.040	0.040	0.039	0.039	0.040	0.039
SE		0.012	0.012	0.010	0.010	0.010	0.010	0.010	0.010	0.010

MLM Level-1 Predictor Only (Fixed and Random Effects)		Mplus (5.1)	MLwiN (2.02)	GLLAMM (Stata 9.2)**	Mplus (5.1)	MLwiN (2.02)	GLLAMM (Stata 9.2)	Mplus (5.1)	MLwiN (2.02)	GLLAMM (Stata 9.2)
Fixed Effects		Unweighted			Weight Method A			Weight Method B		
β_0	(Intercept for MS_UNINS)	0.453	0.453	0.455	0.432	0.432	0.433	0.433	0.433	0.433
SE		0.030	0.030	0.035	0.028	0.028	0.026	0.028	0.028	0.027
β_1	(Slope for Family Income)	-0.089	-0.089	-0.089	-0.080	-0.080	-0.080	-0.080	-0.080	-0.080
SE		0.009	0.009	0.010	0.009	0.008	0.008	0.009	0.009	0.008
Random Effects										
σ^2_ε	Residual Variance (Variation within States)	3.676	3.676	3.676	3.635	3.636	3.635	3.675	2.660	3.675
SE		0.252	0.252	0.254	0.245	0.245	0.248	0.253	0.194	0.255
σ^2_0	Variance in the Intercepts Between States	0.041	0.041	0.041	0.035	0.035	0.035	0.033	0.035	0.033
SE		0.012	0.012	0.015	0.009	0.009	0.007	0.009	0.009	0.008
σ^2_1	Variance in the Slopes Between States	0.003	0.003	0.003	0.003	0.003	0.003	0.003	0.003	0.003
SE		0.001	0.001	0.001	0.001	0.001	0.001	0.001	0.001	0.001
σ_{01}	Covariance	-0.010	-0.010	-0.010	-0.008	-0.008	-0.008	-0.008	-0.008	-0.008
SE		0.004	0.004	0.004	0.002	0.002	0.002	0.002	0.002	0.002

MLM Level-2 Predictor Only		Mplus (5.1)	MLwiN (2.02)	GLLAMM (Stata 9.2)	Mplus (5.1)	MLwiN (2.02)	GLLAMM (Stata 9.2)	Mplus (5.1)	MLwiN (2.02)	GLLAMM (Stata 9.2)
Fixed Effects		Unweighted			Weight Method A			Weight Method B		
β_0	(Intercept for MS_UNINS)	0.453	0.453	0.460	0.456	0.456	0.456	0.457	0.457	0.457
SE		0.027	0.028	0.028	0.027	0.027	0.028	0.027	0.028	0.028
β_1	(Slope for State Poverty)	1.449	1.484	1.449	1.213	1.243	1.213	1.224	1.251	1.224
SE		0.389	0.368	0.393	0.413	0.395	0.418	0.415	0.395	0.457
Random Effects										
σ^2_ε	Residual Variance (Variation within States)	3.752	3.752	3.752	3.703	3.704	3.703	3.742	2.710	3.742
SE		0.263	0.263	0.266	0.254	0.254	0.257	0.262	0.200	0.265
σ^2_0 (τ_{00})	Variance in the Intercepts Between States	0.034	0.034	0.034	0.034	0.034	0.034	0.032	0.034	0.032
SE		0.012	0.012	0.012	0.010	0.010	0.010	0.010	0.010	0.010

MLM Level-1 and Level-2 Predictors		Mplus (5.1)	MLwiN (2.02)	GLLAMM (Stata 9.2)**	Mplus (5.1)	MLwiN (2.02)	GLLAMM (Stata 9.2)	Mplus (5.1)	MLwiN (2.02)	GLLAMM (Stata 9.2)
Fixed Effects		Unweighted			Weight Method A			Weight Method B		
β_0	(Intercept for MS_UNINS)	0.453	0.453	0.454	0.432	0.432	0.433	0.434	0.433	0.434
SE		0.028	0.028	0.026	0.026	0.026	0.025	0.026	0.026	0.026
β_2	(Slope for State Poverty)	1.540	1.583	1.567	1.408	1.436	1.409	1.425	1.453	1.425
SE		0.280	0.284	0.296	0.313	0.304	0.315	0.322	0.304	0.325
β_1	(Slope for Family Income)	-0.090	-0.090	-0.090	-0.080	-0.080	-0.080	-0.080	-0.080	-0.080
SE		0.009	0.009	0.008	0.009	0.009	0.008	0.009	0.009	0.008
Random Effects										
σ^2_ε	Residual Variance (Variation within States)	3.676	3.676	3.676	3.635	3.636	3.635	3.675	2.660	3.675
SE		0.252	0.252	0.026	0.245	0.245	0.248	0.253	0.194	0.255
σ^2_0	Variance in the Intercepts Between States	0.034	0.034	0.040	0.031	0.031	0.031	0.029	0.031	0.029
SE		0.012	0.012	0.017	0.009	0.009	0.007	0.008	0.009	0.008
σ^2_1	Variance in the Slopes Between States	0.003	0.003	0.004	0.003	0.003	0.003	0.003	0.003	0.003
SE		0.001	0.001	0.001	0.001	0.001	0.001	0.001	0.001	0.001
σ_{01}	Covariance	-0.010	-0.010	-0.011	-0.008	-0.008	-0.008	-0.008	-0.008	-0.008
SE		0.004	0.004	0.004	0.003	0.003	0.002	0.003	0.003	0.002

MLM Level-1, Level 2, and Cross-Level Interaction		Mplus (5.1)	MLwiN (2.02)	GLLAMM (Stata 9.2)	Mplus (5.1)	MLwiN (2.02)	GLLAMM (Stata 9.2)	Mplus (5.1)	MLwiN (2.02)	GLLAMM (Stata 9.2)
Fixed Effects		Unweighted			Weight Method A			Weight Method B		
β_0	(Intercept for MS_UNINS)	0.453	0.453	0.453	0.433	0.432	0.433	0.434	0.433	0.434
SE		0.028	0.028	0.028	0.026	0.026	0.024	0.026	0.026	0.025
β_2	(Slope for State Poverty)	1.446	1.478	1.478	1.162	1.204	1.165	1.180	1.217	1.181
SE		0.390	0.369	0.369	0.407	0.389	0.404	0.410	0.389	0.411
β_1	(Slope for Family Income)	-0.090	-0.090	-0.090	-0.080	-0.080	-0.080	-0.080	-0.080	-0.080
SE		0.009	0.009	0.009	0.009	0.008	0.008	0.009	0.009	0.008
β_3	(Slope for Interaction)	0.038	0.042	0.042	0.113	0.108	0.112	0.115	0.111	0.114
SE		0.108	0.115	0.115	0.110	0.131	0.110	0.111	0.131	0.111
σ^2_ε	Residual Variance (Variation within States)	3.676	3.676	3.676	3.635	3.636	3.635	3.675	2.660	3.675
SE		0.252	0.252	0.252	0.245	0.245	0.248	0.253	0.194	0.255
Random Effects										
σ^2_0	Variance in the Intercepts Between States	0.034	0.034	0.034	0.030	0.031	0.031	0.029	0.031	0.029
SE		0.012	0.012	0.012	0.009	0.008	0.007	0.008	0.008	0.008
σ^2_1	Variance in the Slopes Between States	0.003	0.003	0.003	0.003	0.003	0.003	0.003	0.003	0.003
SE		0.001	0.001	0.001	0.001	0.001	0.001	0.001	0.001	0.001
σ_{01}	Covariance	-0.010	-0.010	-0.010	-0.008	-0.008	-0.008	-0.008	-0.008	-0.008
SE		0.004	0.004	0.004	0.003	0.003	0.002	0.003	0.003	0.002
