# Supplementary material for: Fitting multilevel models in complex survey data with design weights: Recommendations
Source: BMC Med Res Methodol. 2009 Jul 14;9:49. doi: 10.1186/1471-2288-9-49 (PMC2717116; doi:10.1186/1471-2288-9-49)
Supplement: Additional file 2 — Categorical outcome parameter and standard error estimates across level-1, level-2, combined-level models, weight scaling methods, and software programs (Mplus estimates a threshold rather than an intercept. These differ only in sign. [21]For presentation, I converted the threshold to a slope). The data provided present the multilevel results across categorical models, weight scaling methods, and software programs. [file 1471-2288-9-49-S2.pdf]

MLM Unconditional Model		Mplus (5.1)	MLwiN (2.02)	GLLAMM (Stata 9.2)	Mplus (5.1)	MLwiN (2.02)	GLLAMM (Stata 9.2)	Mplus (5.1)	MLwiN (2.02)	GLLAMM (Stata 9.2)
Fixed Effects		Unweighted			Weight Method A			Weight Method B		
β_0	(Intercept for MS_UNINS)	-2.433	-2.439	-2.438	-2.403	-2.401	-2.404	-2.398	-2.396	-2.401
SE		0.049	0.049	0.049	0.049	0.050	0.050	0.050	0.051	0.050
Random Effects										
σ^2_0	Variance in the Intercepts Between States	0.104	0.107	0.107	0.111	0.112	0.111	0.108	0.107	0.107
SE		0.023	0.025	0.024	0.026	0.027	0.027	0.026	0.028	0.026
MLM Level-1 Predictor Only (Fixed Effect)		Mplus (5.1)	MLwiN (2.02)	GLLAMM (Stata 9.2)	Mplus (5.1)	MLwiN (2.02)	GLLAMM (Stata 9.2)	Mplus (5.1)	MLwiN (2.02)	GLLAMM (Stata 9.2)
Fixed Effects		Unweighted			Weight Method A			Weight Method B		
β_0	(Intercept for MS_UNINS)	-2.557	-2.561	-2.560	-2.583	-2.584	-2.587	-2.584	-2.579	-2.584
SE		0.049	0.050	0.049	0.051	0.052	0.052	0.051	0.052	0.052
β_1	(Slope for Family Income)	-0.201	-0.201	-0.201	-0.190	-0.190	-0.190	-0.189	-0.190	-0.190
SE		0.010	0.010	0.010	0.011	0.011	0.011	0.012	0.011	0.012
Random Effects										
σ^2_0	Variance in the Intercepts Between States	0.114	0.113	0.113	0.128	0.126	0.127	0.122	0.121	0.122
SE		0.026	0.027	0.026	0.029	0.029	0.029	0.029	0.029	0.029
MLM Level-1 Predictor Only (Fixed and Random Effects)		Mplus (5.1)	MLwiN (2.02)	GLLAMM (Stata 9.2)	Mplus (5.1)	MLwiN (2.02)	GLLAMM (Stata 9.2)	Mplus (5.1)	MLwiN (2.02)	GLLAMM (Stata 9.2)
Fixed Effects		Unweighted			Weight Method A			Weight Method B		
β_0	(Intercept for MS_UNINS)	-2.559	-2.563	-2.563	-2.585	-2.585	-2.589	-2.580	-2.578	-2.584
SE		0.050	0.050	0.050	0.052	0.052	0.052	0.052	0.052	0.052
β_1	(Slope for Family Income)	-0.199	-0.199	-0.199	-0.187	-0.187	-0.187	-0.186	-0.187	-0.187
SE		0.010	0.010	0.010	0.012	0.011	0.012	0.012	0.011	0.012
Random Effects										
σ^2_0	Variance in the Intercepts Between States	0.107	0.107	0.108	0.116	0.115	0.116	0.109	0.108	0.109
SE		0.025	0.025	0.025	0.028	0.028	0.028	0.027	0.029	0.027
σ^2_1	Variance in the Slopes Between States	0.003	0.003	0.003	0.004	0.004	0.004	0.004	0.004	0.004
SE		0.001	0.001	0.001	0.001	0.001	0.001	0.001	0.001	0.001
σ_{01}	Covariance	-0.001	-0.001	-0.001	0.000	0.000	0.000	-0.001	-0.001	-0.001
SE		0.003	0.004	0.004	0.004	0.004	0.004	0.004	0.004	0.004
MLM Level-2 Predictor Only		Mplus (5.1)	MLwiN (2.02)	GLLAMM (Stata 9.2)	Mplus (5.1)	MLwiN (2.02)	GLLAMM (Stata 9.2)	Mplus (5.1)	MLwiN (2.02)	GLLAMM (Stata 9.2)
Fixed Effects		Unweighted			Weight Method A			Weight Method B		
β_0	(Intercept for MS_UNINS)	-2.441	-2.440	-2.439	-2.404	-2.402	-2.404	-2.399	-2.937	-2.401
SE		0.044	0.046	0.046	0.046	0.047	0.047	0.046	0.048	0.047
β_1	(Slope for State Poverty)	2.476	2.440	2.445	2.169	2.154	2.173	2.203	2.181	2.199
SE		0.694	0.814	0.814	0.728	0.729	0.736	0.729	0.730	0.736
Random Effects										
σ^2_0	Variance in the Intercepts Between States	0.088	0.088	0.088	0.097	0.097	0.097	0.092	0.092	0.092
SE		0.023	0.021	0.021	0.024	0.025	0.025	0.024	0.026	0.025
MLM Level-1 and Level-2 Predictors		Mplus (5.1)	MLwiN (2.02)	GLLAMM (Stata 9.2)	Mplus (5.1)	MLwiN (2.02)	GLLAMM (Stata 9.2)	Mplus (5.1)	MLwiN (2.02)	GLLAMM (Stata 9.2)
Fixed Effects		Unweighted			Weight Method A			Weight Method B		
β_0	(Intercept for MS_UNINS)	-2.559	-2.561	-2.561	-2.583	-2.583	-2.586	-2.576	-2.574	-2.579
SE		0.044	0.045	0.045	0.047	0.047	0.047	0.046	0.047	0.047
β_2	(Slope for State Poverty)	3.152	3.148	3.153	3.050	3.040	3.050	3.079	3.068	3.081
SE		0.686	0.796	0.853	0.759	0.737	0.766	0.772	0.745	0.779
β_1	(Slope for Family Income)	-0.197	-0.197	-0.197	-0.186	-0.186	-0.186	-0.184	-0.018	-0.184
SE		0.010	0.010	0.010	0.012	0.011	0.012	0.012	0.011	0.012
Random Effects										
σ^2_0	Variance in the Intercepts Between States	0.082	0.082	0.082	0.091	0.090	0.091	0.084	0.084	0.084
SE		0.023	0.020	0.020	0.025	0.026	0.025	0.024	0.026	0.025
σ^2_1	Variance in the Slopes Between States	0.003	0.003	0.003	0.004	0.004	0.004	0.004	0.004	0.004
SE		0.001	0.001	0.001	0.001	0.001	0.001	0.001	0.001	0.001
σ_{01}	Covariance	-0.005	-0.005	-0.005	-0.006	-0.005	-0.006	-0.007	-0.007	-0.007
SE		0.003	0.003	0.003	0.004	0.003	0.004	0.004	0.003	0.004
MLM Level-1, Level 2, and Cross-Level Interaction		Mplus (5.1)	MLwiN (2.02)	GLLAMM (Stata 9.2)	Mplus (5.1)	MLwiN (2.02)	GLLAMM (Stata 9.2)	Mplus (5.1)	MLwiN (2.02)	GLLAMM (Stata 9.2)
Fixed Effects		Unweighted			Weight Method A			Weight Method B		
β_0	(Intercept for MS_UNINS)	-2.560	-2.563	-2.563	-2.584	-2.585	-2.587	-2.578	-2.576	-2.580
SE		0.044	0.045	0.045	0.047	0.047	0.047	0.046	0.047	0.046
β_2	(Slope for State Poverty)	2.872	2.870	2.874	2.811	2.806	2.814	2.830	2.826	2.832
SE		0.685	0.801	0.800	0.741	0.735	0.747	0.742	0.738	0.749
β_1	(Slope for Family Income)	-0.199	-0.199	-0.199	-0.187	-0.187	-0.187	-0.186	-0.186	-0.186
SE		0.009	0.009	0.009	0.011	0.010	0.011	0.011	0.010	0.011
β_3	(Slope for Interaction)	0.467	0.468	0.467	0.612	0.610	0.612	0.610	0.608	0.610
SE		0.164	0.164	0.164	0.165	0.162	0.167	0.167	0.164	0.169
Random Effects										
σ^2_0	Variance in the Intercepts Between States	0.082	0.082	0.082	0.091	0.090	0.091	0.084	0.083	0.084
SE		0.023	0.020	0.020	0.025	0.026	0.025	0.024	0.026	0.025
σ^2_1	Variance in the Slopes Between States	0.002	0.002	0.002	0.003	0.003	0.003	0.003	0.003	0.003
SE		0.001	0.001	0.001	0.001	0.001	0.001	0.001	0.001	0.001
σ_{01}	Covariance	-0.005	-0.005	-0.005	-0.005	-0.005	-0.005	-0.006	-0.006	-0.006
SE		0.003	0.003	0.003	0.003	0.003	0.003	0.003	0.003	0.003
